# Impaired mitochondrial–endoplasmic reticulum interaction and mitophagy in Miro1-mutant neurons in Parkinson’s disease

**DOI:** 10.1093/hmg/ddaa066

**Published:** 2020-04-13

**Authors:** Clara Berenguer-Escuder, Dajana Grossmann, Paul Antony, Giuseppe Arena, Kobi Wasner, François Massart, Javier Jarazo, Jonas Walter, Jens C Schwamborn, Anne Grünewald, Rejko Krüger

**Affiliations:** 1 Luxembourg Centre for Systems Biomedicine (LCSB), Belvaux, Luxembourg; 2 Section for Translational Neurodegeneration “Albrecht Kossel”, Department of Neurology, Universitätsmedizin Rostock, Rostock, Germany; 3 Institute of Neurogenetics, University of Lübeck, Lübeck, Germany; 4 Parkinson Research Clinic, Centre Hospitalier de Luxembourg (CHL) , Luxembourg City, Luxembourg; 5 Transversal Translational Medicine, Luxembourg Institute of Health (LIH), Strassen, Luxembourg

## Abstract

Mitochondrial Rho GTPase 1 (Miro1) protein is a well-known adaptor for mitochondrial transport and also regulates mitochondrial quality control and function. Furthermore, Miro1 was associated with mitochondrial-endoplasmic reticulum (ER) contact sites (MERCs), which are key regulators of cellular calcium homeostasis and the initiation of autophagy. Impairments of these mechanisms were linked to neurodegeneration in Parkinson’s disease (PD). We recently revealed that PD fibroblasts harboring Miro1 mutations displayed dysregulations in MERC organization and abundance, affecting mitochondrial homeostasis and clearance. We hypothesize that mutant Miro1 impairs the function of MERCs and mitochondrial dynamics, altering neuronal homeostasis and integrity in PD. PD skin fibroblasts harboring the Miro1-R272Q mutation were differentiated into patient-derived neurons. Live-cell imaging and immunocytochemistry were used to study mitophagy and the organization and function of MERCs. Markers of autophagy or mitochondrial function were assessed by western blotting. Quantification of organelle juxtapositions revealed an increased number of MERCs in patient-derived neurons. Live-cell imaging results showed alterations of mitochondrial dynamics and increased sensitivity to calcium stress, as well as reduced mitochondrial clearance. Finally, western blot analysis indicated a blockage of the autophagy flux in Miro1-mutant neurons. Miro1-mutant neurons display altered ER-mitochondrial tethering compared with control neurons. This alteration likely interferes with proper MERC function, contributing to a defective autophagic flux and cytosolic calcium handling capacity. Moreover, mutant Miro1 affects mitochondrial dynamics in neurons, which may result in disrupted mitochondrial turnover and altered mitochondrial movement.

## Introduction

Parkinson’s disease (PD) is a chronic neurodegenerative disorder, the cause of which currently remains elusive (1). Most cases of PD are idiopathic and only ~10% are caused by genetic mutations (2). To date, 23 loci have been identified, in which mutations are proposed to cause PD (3), and >20 risk factors are suggested to be involved in the pathogenesis of the disorder (4).

The key histopathological hallmark of PD is the degeneration of dopaminergic (DA) neurons in the substantia nigra pars compacta (5). The molecular mechanisms underlying the neuronal cell death are still under debate. However, animal- and patient-based cellular models of genetic PD forms help identify common molecular pathways involved in the pathogenesis of PD (6,7).

Mitochondria are highly dynamic organelles that act as crucial players in maintaining cellular function and homeostasis. Mitochondrial dynamics and quality control are key pathways in the pathogenesis of familial and sporadic PD (8–10). DA neurons are considered to be particularly susceptible to mitochondrial dysfunction compared with other types of neurons, since they require large amounts of energy to maintain the integrity of their long axons, regulate their pace-making activity and control intrinsic calcium oscillations (11,12). In this regard, mitochondrial-endoplasmic reticulum (ER) contact sites (MERCs) recently came into focus in the context of PD research as one of the potential mechanisms underlying the pathogenesis of the disease. Transfer of calcium and lipids at MERCs is required for the maintenance of mitochondrial energy production and membrane stability (13,14). Several PD-associated proteins were reported to regulate the function and number of MERCs, such as Parkin (15,16), DJ-1 (17), α-synuclein (18,19) and PINK1 (16,20,21), suggesting that these contacts may play a crucial role in neurodegeneration.

The mitochondrial Rho GTPase Miro1 is an adaptor protein for the transport of mitochondria (22–25). Furthermore, Miro1 emerged as an important regulator of mitochondrial calcium homeostasis (26,27), mitochondrial dynamics (28,29) and PINK1/Parkin-mediated mitochondrial quality control (30–33). In addition, Miro1 was recently shown to directly interact with the PD-associated proteins LRRK2 and α-synuclein. In the presence of mutations in these proteins, the interaction with Miro1 is impaired, stabilizing Miro1 on the outer membrane of dysfunctional mitochondria and, thereby, delaying the arrest of mitochondrial transport for mitophagy (34,35). Interestingly, Miro1 orthologues Gem1 and dMiro (yeast and *Drosophila*, respectively) have been shown to localize to MERCs in a similar fashion as mammalian Miro1 (36,37), suggesting a potential role for Miro1 as a key regulator of the mitochondria–ER interface.

We have previously shown that PD-related mutations in Miro1 affect the amount of MERCs in patient-derived fibroblasts (38), demonstrating the importance of Miro1 in the regulation of ER–mitochondria crosstalk in PD. To develop a closer-to-disease patient-based cellular model, we used these fibroblasts to generate induced-pluripotent stem cell (iPSC)-derived Miro1-R272Q mutant neurons, and we provide the first insights into the pathogenic effect of PD-associated mutant Miro1 in human midbrain neurons.

## Results

### Miro1 protein levels and mitochondrial mass are unaffected by Miro1-R272Q in neurons

**Figure 1:**
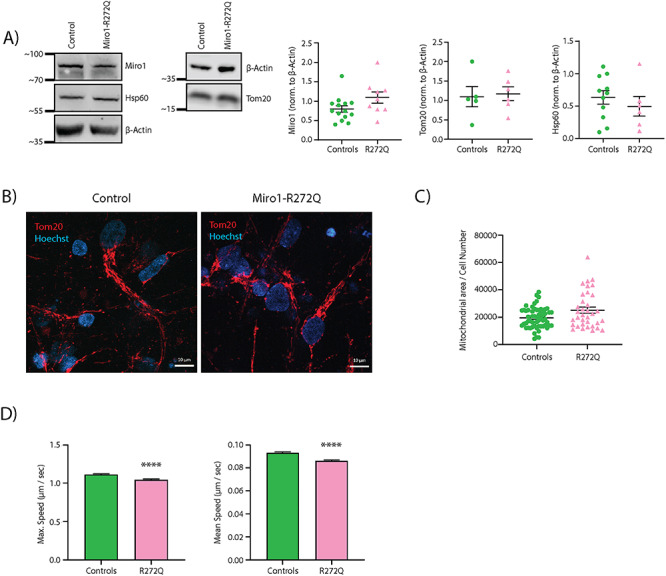
Miro1-R272Q does not affect mitochondrial mass, but reduces mitochondrial movement. (**A**) Representative western blot images of Miro1 protein and the mitochondrial marker proteins Tom20 and Hsp60 in iPSC-derived neurons. Corresponding densitometries of western blot analyses normalized to β-actin are shown at the right. Data indicated as mean ± SEM (*n* = 3). (**B**) iPSC-derived neurons were fixed and stained against Tom20. Images were obtained using a 63× objective; scale bars indicate 10 μm. (**C**) Quantification of mitochondrial area per cell from the Tom20 signal from images shown in B. Data indicated as mean ± SEM. Significance calculated by Mann–Whitney test (*n* = 4). (**D**) Analysis of maximum and mean speed per mitochondria from iPSC-derived neurons stained with MitoTracker Green FM. Data indicated as mean ± SEM. Significance calculated by Mann–Whitney test; ^*^^*^^*^P < 0.001; (*n* = 5).

Based on our previous discovery that Miro1-mutant fibroblasts expressed decreased levels of Miro1 protein and mitochondrial mass (38), we first quantified relative levels of Miro1 and the mitochondrial markers Tom20 and Hsp60. Interestingly, Miro1 levels were not significantly different between mutant and control neurons (Fig. 1A), indicating that the R272Q mutation does not have an impact on Miro1 protein stability in iPSC-derived neurons. Moreover, neither Tom20 nor Hsp60 protein levels were changed in patient-derived neurons compared with control neurons, suggesting no differences in mitochondrial mass between cell lines (Fig. 1A). In addition, quantification of Tom20 area by immunocytochemistry confirmed that mitochondrial mass was not affected in Miro1-R272Q mutant neurons (Fig. 1B and C).

### Mitochondrial movement is altered in Miro1-R272Q mutant iPSC-derived neurons

It is well established that the dysfunction of Miro1 disturbs the mitochondrial transport in neurons (26,39). Moreover, recent studies suggested that Miro1-mediated mitochondrial transport is especially important in PD, since human skin fibroblasts and iPSC-derived neurons expressing *LRRK2* or *SNCA* mutations cause a stabilization of Miro1 on damaged mitochondria, interfering with their detachment from the transport machinery and, consequently, prohibiting mitophagy (34,35,40). Thus, we were interested in assessing mitochondrial movement in Miro1-R272Q mutant neurons. We used the mitochondrial dye MitoTracker Green FM to analyze mitochondrial velocity in neuronal projections. Miro1-R272Q mutant neurons showed significantly decreased mean and maximum mitochondrial speed in comparison with control neurons (Fig. 1D), which is not related to alterations of Miro1 protein levels.

### Impaired cytosolic calcium handling in Miro1-R272Q mutant iPSC-derived neurons

**Figure 2:**
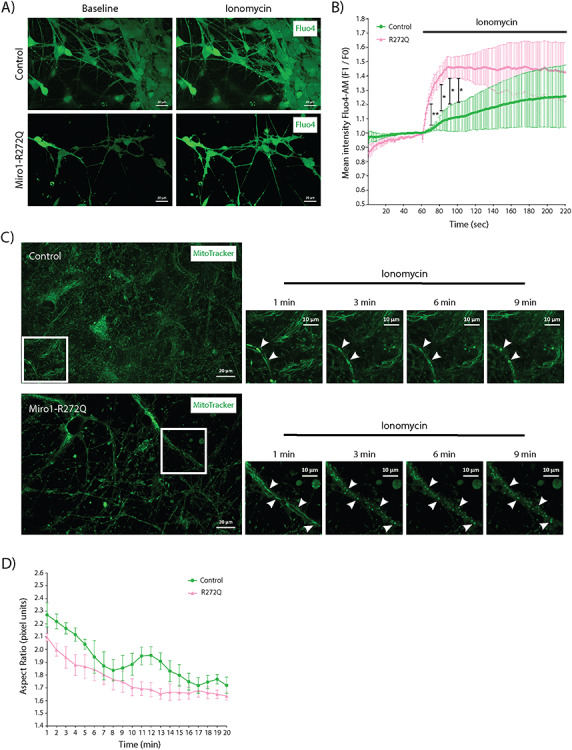
Miro1-R272Q impairs cytosolic calcium handling and increases sensitivity to calcium stress*.* (**A**) iPSC-derived neurons were loaded with the cytosolic calcium indicator Fluo-4 AM for live-cell imaging. During imaging, cells were treated with 5 μM ionomycin in order to increase cytosolic calcium levels. Imaging was continued for 10 min with a 2 s interval. Images were obtained with a 40× objective. (**B**) Analysis of mean Fluo-4 AM fluorescence intensity (F1/F0) from images shown in A. Data indicated as mean ± SEM. Significance of first time points after treatment calculated by Holm–Sidak multiple *t* test; ^*^P < 0.05; ^*^^*^P < 0.01; (*n* = 4–6). (**C**) iPSC-derived neurons were stained with MitoTracker Green FM for live-cell imaging. Images were obtained once per minute using a 40× objective. During imaging, neurons were treated with 5 μM ionomycin and mitochondrial morphology was analyzed using ImageJ. The white boxes in the images indicate the magnified regions showing representative time points during the treatment. Scale bars indicate 20 μm in the large images, and 10 μm in the magnified images. (**D**) Analysis of mitochondrial fragmentation in control and mutant neurons, expressed as aspect ratio, from images shown in C. Data indicated as mean ± SEM. Significance calculated by Holm–Sidak multiple *t* test (*n* = 4–5).

Previous studies have linked changes of mitochondrial motility to mitochondrial calcium levels, showing that the lower the calcium content in the mitochondrial matrix, the faster the movement of the mitochondria (27,41). We previously showed that mitochondria from Miro1-mutant fibroblasts displayed a decreased capacity to buffer calcium after thapsigargin treatment (38). To explore how Miro1-R272Q mutant neurons respond to cytosolic calcium increase, we quantified the fluorescent signal of the cytosolic calcium indicator Fluo4-AM after treatment with the calcium ionophore ionomycin. As expected, both control and patient-derived neurons increased cytosolic calcium levels in response to ionomycin exposure. However, while control neurons displayed a relatively low calcium peak, Miro1-R272Q mutant neurons showed a high peak of cytosolic calcium after treatment (Fig. 2A and B), suggesting a decreased capacity to handle changes of cytosolic calcium levels.

Miro1 acts as a cytosolic calcium sensor, mediated by the two calcium-binding EF-hand domains (42,43). A recent study showed that Miro1 is required to induce mitochondrial fragmentation in the presence of elevated cytosolic calcium levels, a mechanism defined as mitochondrial shape transition (MiST) (44). Moreover, Nemani and colleagues identified MiST as a prerequisite for the initiation of mitophagy. To explore this mechanism in a PD-related Miro1-mutant background, iPSC-derived neurons were stained with MitoTracker Green FM and treated with ionomycin during live-cell imaging. All neuron lines reacted to the ionomycin exposure with calcium-mediated mitochondrial fragmentation. Similar to Miro1-mutant fibroblasts (38), Miro1-R272Q mutant neurons showed a quicker mitochondrial fragmentation, reflected by a faster decline of the aspect ratio compared with control neurons (Fig. 2C and D), although these differences did not reach statistical significance.

### Increased MERCs in Miro1-R272Q mutant iPSC-derived neurons

**Figure 3:**
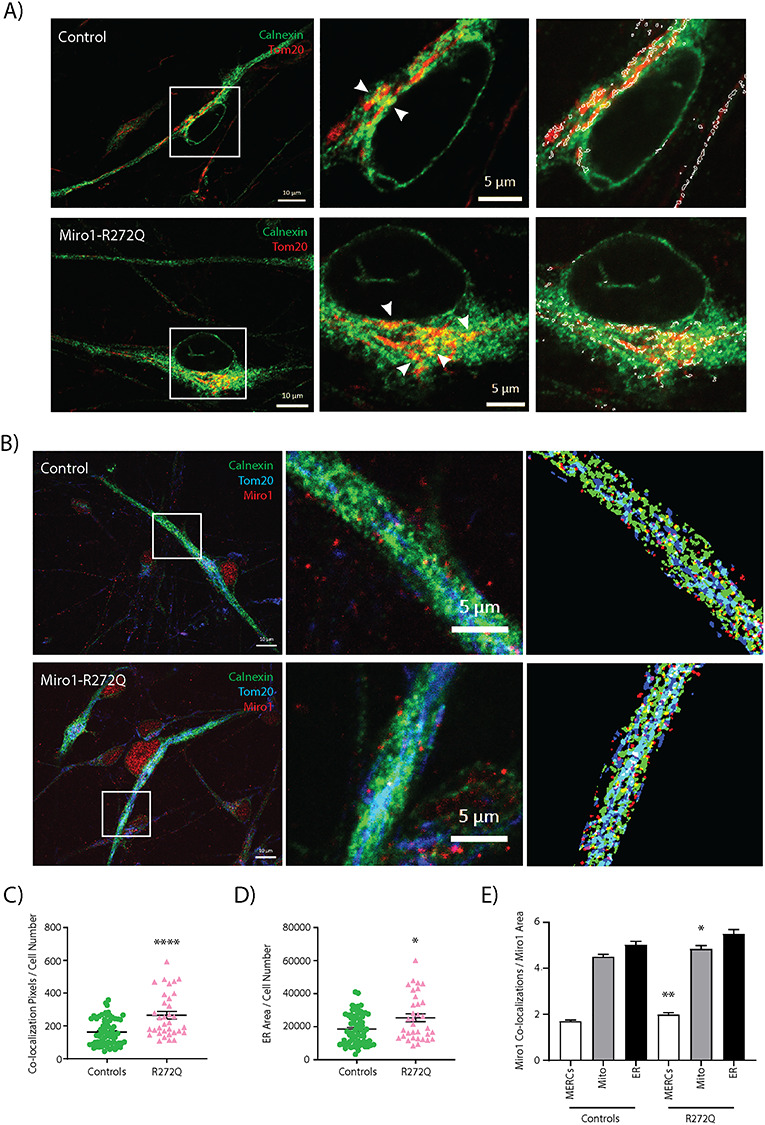
Miro1-R272Q causes increased MERCs and increased localization of Miro1 to MERCs. (**A**) iPSC-derived neurons were fixed and stained with antibodies against the ER marker protein Calnexin and the mitochondrial marker protein Tom20. Images were obtained using a 63× objective; scale bars indicate 10 and 5 μm. Every field consisted of z-stacks of 0.5 μm interval. The white boxes in the merged images indicate the co-localization panels and the magnified regions where co-localizations are indicated with white arrows. (**B**) iPSC-derived neurons were fixed and stained with antibodies against Calnexin, Tom20 and Miro1. Images were obtained using a 63× objective; scale bars indicate 10 μm in the large images, and 5 μm in the magnified images. The white boxes in the merged images shown at the left indicate the magnified regions and the co-localization panels shown at the right. Co-localization of Miro1 signal with MERCs is indicated as white labels in the co-localization panels. (**C**) Quantification of co-localization events of Calnexin and Tom20 per cell, indicating the amount of MERCs, from images shown in A. (**D**) Quantification of ER area per cell from the Calnexin signal from images shown in A. (**E**) Quantification of co-localization events of Miro1 puncta with MERCs, mitochondria or the ER per cell from images shown in B. All data indicated as mean ± SEM. Significance calculated by Mann–Whitney test; ^*^P < 0.05; ^*^^*^^*^P < 0.001; (*n* = 4).

Miro1 is implicated in the regulation of MERCs (36,45), which are crucially involved in cellular calcium homeostasis (14,37). Since we previously observed reduced number of juxtapositions between ER and mitochondria in Miro1-mutant fibroblasts (38), we next analyzed MERCs in Miro1-R272Q mutant neurons by quantifying the co-localization of both organelles after immunostaining of the ER and mitochondrial marker proteins Calnexin and Tom20, respectively (Fig. 3A). The analysis revealed a significantly increased ER-mitochondria co-localization in Miro1-R272Q mutant neurons compared with control neurons (Fig. 3C). Remarkably, we also noticed an overall increase of the ER mass in patient-derived neurons (Fig. 3D).

Next, we tested whether the observed quantitative differences in MERCs was accompanied by an altered localization of mutant Miro1 at the different subcellular compartments. Therefore, we performed a triple immunostaining against Tom20, Calnexin and Miro1 (Fig. 3B). Interestingly, when adjusting for baseline Miro1 levels, we detected a significantly increased amount of Miro1 puncta juxtaposed to MERCs and mitochondria in Miro1-R272Q mutant neurons compared with control neurons (Fig. 3E). These data suggest that the R272Q mutation may influence the interaction of Miro1 with other proteins residing in MERCs, leading to alterations of the amount of contact sites.

### Mitophagy flux is blocked in Miro1-R272Q mutant iPSC-derived neurons

Previous publications described the PINK1/Parkin-mediated removal of Miro1 by the proteasome as part of the initial step for mitochondrial clearance (32,33,46). Moreover, it was suggested that proteins involved in mitophagy initiation (i.e. Mfn2) may also act as mitochondria-ER tethers (46), and when Parkin targets these proteins for degradation, mitochondria are untethered from the ER to facilitate mitophagy (47). Therefore, we hypothesize that the increased amount of MERCs identified in Miro1-R272Q mutant neurons physically interferes with mitochondrial clearance.

**Figure 4:**
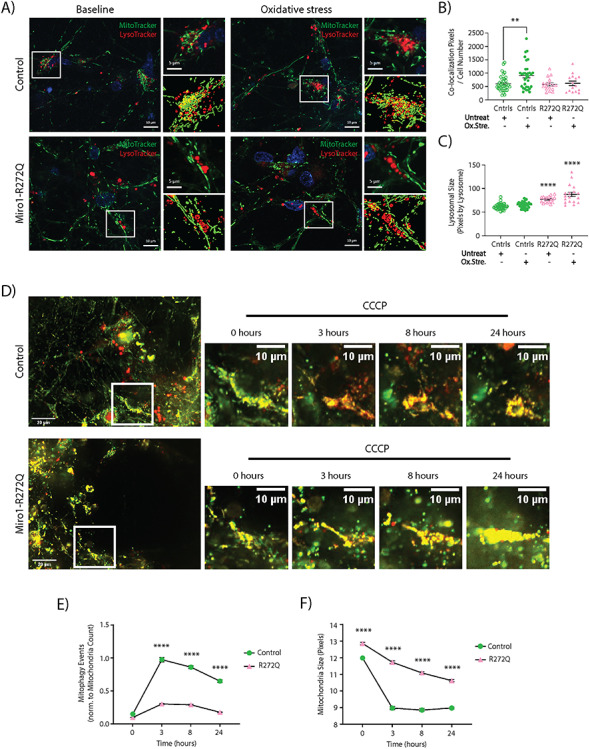
Miro1-R272Q mutant neurons show decreased mitophagy flux and mitochondrial fragmentation. (**A**) iPSC-derived neurons were stained with MitoTracker Green FM and LysoTracker Deep Red for live-cell imaging. Images were obtained using a 63× objective, scale bars indicate 10 μm in the large images and 5 μm in the magnified images. Every field consisted in z-stacks of 0.5 μm interval. The white boxes in the merged images indicate the magnified regions and co-localization panels shown at the right. Co-localization events of MitoTracker and LysoTracker signals are indicated as yellow labels in the co-localization panels. (**B**) Quantification of co-localization events of MitoTracker and LysoTracker per cell, indicating the amount of mitophagy events, from images shown in A. Data indicated as mean ± SEM. Significance calculated by Mann–Whitney test; ^*^^*^P < 0.01; ^*^^*^^*^P < 0.001; (*n* = 3–6). (**C**) Analysis of the mean size of individual Lysotracker structures in pixels, indicating the mean lysosomal size, from images shown in A. Data indicated as mean ± SEM. Significance calculated by Mann–Whitney test; ^*^^*^P < 0.01; ^*^^*^^*^P < 0.001; (*n* = 3–6). (**D**) Gene-edited iPSC-derived neurons expressing the ATP5C1-Rosella reporter (Mitorosella) were treated with 10 μM CCCP to induce mitophagy. The white boxes in the images on the left indicate the magnified regions showing representative time points during the treatment. Scale bars indicate 20 μm in the images of the left, and 10 μm in the magnified images. (**E**) Analysis of the mean size of individual mitochondria in pixels from images shown in D. Data indicated as mean ± SEM. Significance calculated by Bonferroni multiple comparisons test; ^*^^*^^*^P < 0.001; (*n* = 4). (**F**) Quantification of only-red particles normalized to mitochondria count, indicating the amount of mitophagy events, from images shown in D. Data indicated as mean ± SEM. Significance calculated by Bonferroni multiple t test; ^*^^*^^*^P < 0.001; (*n* = 4).

To induce mitophagy, we cultured control and Miro1-R272Q mutant neurons with antioxidant-free medium for 24 h to induce mild oxidative stress. Next, we stained these neurons with MitoTracker Green FM and LysoTracker Deep Red to analyze mitochondria and lysosome co-localization by live-cell imaging as a mitophagy readout (48,49) (Fig. 4A). After induction of mild oxidative stress, a significantly increased co-localization of mitochondria and lysosomes was observed in control neurons, but not in Miro1-R272Q mutant neurons (Fig. 4B). Remarkably, we also noticed that patient-derived neurons showed significantly larger lysosomes compared with control neurons (Fig. 4C).

To further investigate the observed impairment of mitochondrial quality control, we monitored mitophagy over time in living neurons by using the ATP5C1-Rosella fluorescent protein biosensor construct (Mitorosella) (50). The Mitorosella reporter is formed by a tandem of the fluorescent proteins DsRed and pH sensor pHluorin combined with the open reading frame of ATP5C1, which functions as a mitochondrial targeting sequence. The signal of pHluorin, acting as a modified GFP, is quenched in acidic environments (like the autophagolysosome lumen), whereas the DsRed fluorescent signal remains visible. Therefore, ‘non-mitophagy events’ are represented as healthy mitochondria expressing both fluorescent signals at the same time, while ‘mitophagy events’ are represented as mitochondria engulfed by the autophagolysosome expressing only DsRed fluorescence (50).

iPSC-derived neurons expressing the Mitorosella reporter were treated with carbonyl cyanide m-chlorophenyl hydrazine (CCCP) for 24 h to induce mitochondrial depolarization and subsequent degradation (51). In control neurons, a significantly increased number of mitophagy events was observed after 3 h of CCCP treatment (Fig. 4D and E). These mitophagy events decreased over time, indicating the complete elimination of engulfed mitochondria and the recovery of control neurons from the perturbation. As expected, Miro1-R272Q mutant neurons show decreased mitochondrial clearance after CCCP treatment (Fig. 4D and E). Intriguingly, when analyzing mitochondrial morphology, Miro1-R272Q mutant neurons showed delayed CCCP-induced mitochondrial fragmentation compared with control neurons (Fig. 4F). Taken together, these results suggest that the excess of MERCs observed in Miro1-R272Q mutant neurons might interfere with CCCP-induced mitochondrial fragmentation and the initiation of mitophagy.

### Autophagy flux is blocked in Miro1-R272Q mutant iPSC-derived neurons

**Figure 5:**
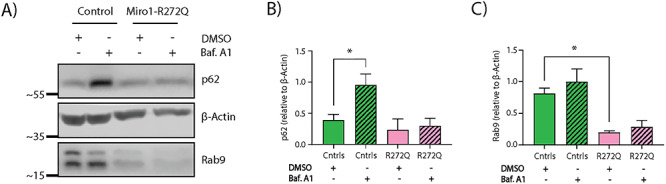
Miro1-R272Q leads to alterations of autophagy flux and Rab9 protein levels. (**A**) Representative western blot image of p62 and Rab9 proteins in iPSC-derived neurons after bafilomycin A1 treatment. (**B)** Densitometry of p62 western blot analysis normalized to β-actin. Data indicated as mean ± SEM. Significance calculated by Mann–Whitney test; ^*^P < 0.05; (*n* = 3). (**C**) Densitometry of Rab9 western blot analysis normalized to β-actin. Data indicated as mean ± SEM. Significance calculated by Mann–Whitney test; ^*^P < 0.05; (*n* = 3).

Accumulating evidence suggests that MERCs are involved in the initiation of autophagy by the recruitment of proteins required for autophagosome generation (52–54). Considering the increased amount of MERCs and impaired mitophagy flux in Miro1-R272Q mutant neurons, we investigated whether the general autophagy flux was also affected. To study the ATG5/7-dependent autophagy pathway, we treated control and patient-derived neurons for 24 h with bafilomycin A1, an inhibitor of the lysosomal degradation pathway. Western blotting results showed that levels of the autophagosome-associated protein p62 were increased in control neurons after treatment, but not in Miro1-R272Q mutant neurons (Fig. 5A and B), suggesting an inhibition of the canonical autophagy pathway.

In addition to these results, we investigated an alternative ATG5/7-independent autophagy pathway, in which autophagosome generation is mediated by Rab9 GTPase at the Golgi apparatus (55–57). In our previous study, we showed that Miro1-mutant fibroblasts treated with bafilomycin A1 displayed increased accumulation of Rab9, but not of the ATG5/7-dependent autophagy marker LC3II (38). In contrast, we did not detect any significant increase of Rab9 in mutant neurons after bafilomycin A1 treatment compared with the untreated condition (Fig. 5A and C). Moreover, Miro1-R272Q mutant neurons also displayed decreased baseline levels of Rab9 compared with control neurons (Fig. 5A and C).

## Discussion

Although several lines of evidence support a role for Miro1 in neurodegeneration, PD-associated genetic variants in the *RHOT1* gene have only recently been identified (38). Expanding our previous research in patient-derived fibroblasts, we now explored the pathogenic mechanisms of PD-related mutant Miro1 in iPSC-derived neurons. Our findings, together with other studies, further highlight the importance of Miro1 function in neurodegeneration, in particular in the pathogenesis of PD.

It was reported that mutations within the EF-hand domains of Gem1, the yeast ortholog of Miro1, interfered with the calcium-binding function of the protein, causing the instability and subsequent degradation of the mutant protein *in vivo* (58). Levels of Miro1 protein might also be affected by increased mitochondrial turnover, as we and others already demonstrated (38,46,59). In contrast, basal levels of Miro1 protein, in addition to basal levels of the mitochondrial markers Tom20 and Hsp60, in Miro1-R272Q mutant neurons were unchanged compared with control neurons. These results indicate that, in patient-derived neurons, baseline levels of mutant Miro1 are not influenced neither by the R272Q mutation nor by mitochondrial clearance. A plausible explanation might be that post-mitotic neurons, unlike mitotic cells such as fibroblasts, are unable to tolerate extensive removal of mitochondria or big changes of mitochondrial distribution to meet their high energy demands (60).

The role of Miro1 in promoting mitochondrial movement along the cytoskeleton is well established (22,24,25). In particular, Miro1-mediated regulation of mitochondrial transport is of crucial importance for neuronal survival, as dysregulations were shown to interfere with mitophagy in different cellular models of PD (34,35). Here, we observed a decrease in velocity of moving mitochondria in Miro1-R272Q mutant neurons, suggesting that the R272Q mutation affects the regulation of mitochondrial speed, which is supported by other studies (26,39).

Remarkably, reduction of mitochondrial motility was linked to mitochondrial calcium uptake from the cytosol (41), a process that appears to be modulated by the interaction of Miro1 and the mitochondrial calcium uniporter (MCU) (27). Here, we could demonstrate that neurons derived from a PD patient harboring the R272Q mutation in Miro1 also show altered cytosolic calcium handling when treated with ionomycin, which is in line with our previous findings in patient-derived fibroblasts (38). Intriguingly, our analysis of calcium-induced mitochondrial fragmentation revealed that mutant iPSC-derived neurons display a moderately higher sensitivity to calcium stress. The R272Q mutation is localized within one of the two EF-hand domains of Miro1 (38), which are involved in the initiation of calcium-induced mitochondrial fragmentation (44). Therefore, we hypothesize that the calcium-sensing function of Miro1 is disrupted by the R272Q mutation.

Mammalian Miro1 was found to localize to MERCs (36,45), and MERCs were described to regulate cytosolic and mitochondrial calcium homeostasis (37,61). Several studies demonstrated that an altered number of MERCs led to impaired mitochondrial calcium buffering (14,15). In line with these findings, our experiments indicated an increase in the abundance of MERCs in Miro1-mutant neurons at baseline. Although Miro1-mutant fibroblasts displayed a reduced general amount of MERCs, we speculate that these differences might be cell type-specific, since Parkin-mutant mitotic cells exhibited a decrease of MERCs (15,62), while PINK1-, Parkin- or Miro1-knockdown neurons displayed increased MERCs (14,16). Moreover, we detected increased levels of Miro1 puncta at MERCs in patient-derived neurons, suggesting that the mutant protein may stabilize these organelle contact sites in a pathogenic fashion.

In addition to their role in managing intracellular calcium levels, MERCs were shown to act as regulators of mitophagy in neurons, given that the untethering of the mitochondria from the ER is required for the progression of mitochondrial quality control (47). In line with these findings, treatment of Miro1-mutant neurons with CCCP caused an inhibition of mitochondrial clearance and a delay in mitochondrial fragmentation, which was proposed as a mechanism that precedes mitophagy (46,51,63). These data support our hypothesis that the R272Q mutation in Miro1 not only enhances mitochondria and ER tethering, but also interferes with the function of MERCs in regulating calcium homeostasis and initiation of mitophagy.

MERCs are likewise involved in the initiation of autophagy in terms of ER-derived autophagosome formation (52,54,64). Miro1-R272Q mutant neurons show no accumulation of p62 protein levels after blockage of lysosomal degradation, indicating a possible inhibition of the autophagy pathway. We and others have previously illustrated that Rab9-driven autophagy can substitute the canonical ATG5/7-dependent autophagy pathway in skin fibroblasts (38,55) and in cardiomyocytes (56,57). However, we could not detect an increase of Rab9 protein levels in Miro1-R272Q mutant neurons upon bafilomycin A1 treatment, suggesting that ATG5/7-independent autophagy does not play a compensatory role in patient-derived neurons. In addition, baseline levels of Rab9 protein were already significantly lower in patient-derived neurons compared with control neurons. In the current study, the functional link between Miro1 and Rab9 was not further assessed, but follow-up experiments are planned iln the future.

In summary, here, we describe how PD-associated mutant Miro1 is involved in the pathogenesis of the disease by using iPSC-derived neurons differentiated from previously characterized patient-derived fibroblasts. Mutant Miro1 affects the movement of mitochondria along the cytoskeleton and stabilizes MERCs. In turn, the physiological functions of Miro1 in mitochondrial calcium handling, mitophagy and autophagy initiation are disrupted. Solving how Miro1 regulates these cellular processes will be crucial for the design of Miro1-modulating therapies that target neurodegeneration in PD.

## Materials and Methods

### Generation and characterization of iPSCs from skin fibroblasts

Human skin fibroblasts from PD patients with mutations in Miro1 were obtained as previously described (38). Reprogramming of control skin fibroblasts was achieved using the CytoTune-iPS 2.0 Sendai Reprogramming Kit (Thermo Fisher Scientific, A16517). Patient-derived fibroblasts harboring the heterozygous mutation Miro1-R272Q were reprogrammed using synthetic modified mRNA (65). iPSC colonies were expanded in culture and maintained with Essential 8 medium (Thermo Fisher Scientific, A1517001) supplemented with 1% penicillin-streptomycin (Thermo Fisher Scientific, 15140163).

Pluripotency markers of iPSC lines were validated by immunostaining and flow cytometry using the LSRFortessa™ cell analyzer (BD Biosciences) (Supplementary Material, Fig. S1A and B). For that purpose, antibodies against Sox2 (Santa Cruz Biotechnologies, sc-17 320, RRID:AB_2286684, dilution 1:200; secondary antibody: donkey anti-goat Alexa Fluor 647, Life Technologies, A-21447, RRID:AB_141844, dilution 1:1000), Nanog (Abcam, ab21624, RRID:AB_446437, dilution 1:250; secondary antibody: goat anti-rabbit Alexa Fluor 568, Life Technologies, A-11011, RRID:AB_143157, dilution 1:1000), Oct4 (Santa Cruz Biotechnologies, sc-5279, RRID:AB_628051, dilution 1:1000; secondary antibody: goat anti-mouse Alexa Fluor 488, Life Technologies, A-11001, RRID:AB_2534069, dilution 1:1000), SSEA3 (DSHB, MC-631, RRID:AB_528476, dilution 1:200; secondary antibody: goat anti-rat Alexa Fluor 568, Life Technologies, A-11077, RRID:AB_141874, dilution 1:1000) and Tra-160 (Abcam, ab16288, RRID:AB_778563, dilution 1:200; secondary antibody: goat anti-mouse Alexa Fluor 488, Life Technologies, A-11001, RRID:AB_2534069, dilution 1:1000) were used. The ability of the iPSC lines to differentiate into cells of the three germ layers was verified using the Human Pluripotent Stem Cell Functional Identification Kit (R&D Systems, SC027) (Supplementary Material, Fig. S1C).

### Neuronal differentiation and characterization of iPSC-derived neurons

iPSCs were differentiated into small molecule neural precursor cells (smNPCs), as previously published (66). smNPCs were expanded in culture and maintained with N2B27 medium consisting of 50% Neurobasal medium (Thermo Fisher Scientific, 21103-049) and 50% DMEM-F12 medium (Thermo Fisher Scientific, 11320-033), supplemented with 0.5% N2 (Thermo Fisher Scientific, 17502-048), 1% B27 (Thermo Fisher Scientific, 17504-044), 1% Glutamax (Thermo Fisher Scientific, 35050-061) and 1% penicillin-streptomycin (Thermo Fisher Scientific, 15140163). smNPC identity was verified via immunostaining of neuronal precursor markers (Supplementary Material, Fig. S2A). For that purpose, antibodies against Musashi (Abcam, ab21628, RRID:AB_2144988, dilution 1:250; secondary antibody: goat anti-rabbit Alexa Fluor 488, Life Technologies, A-11008, RRID:AB_143165, dilution 1:1000), Nestin (R&D Systems, MAB1259, RRID:AB_2251304, dilution 1:250; secondary antibody: goat anti-mouse Alexa Fluor 647, Life Technologies, A-21235, RRID:AB_2535804, dilution 1:1000), Sox1 (R&D Systems, AF3369, RRID:AB_2239879, dilution 1:250; secondary antibody: donkey anti-goat Alexa Fluor 647, Life Technologies, A-21447, RRID:AB_141844, dilution 1:1000) and Sox2 (Santa Cruz Biotechnologies, sc-17 320, RRID:AB_2286684, dilution 1:250; secondary antibody: donkey anti-goat Alexa Fluor 647, Life Technologies, A-21447, RRID:AB_141844, dilution 1:1000) were used.

Further neuronal differentiation from smNPCs was performed according to the protocol described previously (66). Neurons were maintained until day 20–30 of maturation and then used for imaging experiments. Neuronal characterization by immunostaining was performed to confirm successful differentiation (Supplementary Material, Fig. S2B and C). For that purpose, antibodies against TH (Millipore, AB152, RRID:AB_390204, dilution 1:500; secondary antibody: goat anti-rabbit Alexa Fluor 568, Life Technologies, A-11011, RRID:AB_143157, dilution 1:1000), Tuj1 (Covance, MMS-435P, RRID:AB_2313773, dilution 1:500; secondary antibody: goat anti-mouse Alexa Fluor 488, Life Technologies, A-11001, RRID:AB_2534069, dilution 1:1000) and FoxA2 (Santa Cruz Biotechnologies, sc-101 060, RRID:AB_1124660, dilution 1:500; secondary antibody: goat anti-mouse Alexa Fluor 488, Life Technologies, A-11001, RRID:AB_2534069, dilution 1:1000) were used.

### SDS-PAGE and western blotting

Lysates from iPSC-derived neurons were prepared in RIPA buffer containing 1× complete protease inhibitor (Roche complete, Roche). Proteins were subsequently separated by electrophoresis on acrylamide gels and transferred to nitrocellulose membranes, as previously described (38). Antibodies against Miro1 (Sigma Aldrich, WH0055288M1, RRID:AB_1843347), Tom20 (Santa Cruz Biotechnologies, sc-17 764, RRID:AB_628381), Hsp60 (Cell Signaling, 4870S, RRID:AB_2295614), Rab9 (Santa Cruz Biotechnologies, sc-74 482, RRID:AB_1128898), p62 (BD Transduction, 610 833, RRID:AB_398152) and β-Actin (Thermo Fisher Scientific, MA1-744, RRID:AB_2223496) were used. Densitometry analysis of western blot membranes was performed using the ImageJ software (Wayne Rasband; National Institutes of Health, USA).

### Live-cell imaging

iPSC-derived neurons were seeded on Nunc™ Lab-Tek™ Chamber slides (Thermo Fisher Scientific, 154534PK). Live-cell imaging was performed using a Live Cell Microscope Axiovert 2000 with spinning disc, plan-apochromate objectives and Hamamatsu camera C11440 (Carl Zeiss Microimaging GmbH, Jena, Germany).

For mitochondrial movement analysis, neurons were stained with 100 nM MitoTracker® Green FM (Thermo Fisher Scientific, M7514) for 50 min at 37°C to visualize mitochondria at day 20 of neuronal differentiation. Time-lapse videos were obtained for 10 min with a 1 s interval. Mitochondrial movements were subsequently analyzed with the TrackMate plugin of ImageJ (67).

For mitochondria-lysosome co-localization studies, neurons were stained at day 30 of differentiation with 100 nM MitoTracker Green FM and LysoTracker™ Deep Red (Thermo Fisher Scientific, L12492) for 20 min at 37°C to visualize mitochondria and lysosomes, respectively. Both fluorescent channels were acquired simultaneously. Images were acquired using 500 nm z-steps between planes.

For calcium imaging, neurons were stained with Fluo4-AM (Thermo Fisher Scientific, F10489) at day 30 of neuronal differentiation. During imaging, Fluo4-AM was present in the medium at a 50% dilution. During imaging, cells were treated with 5 μM ionomycin (Sigma-Aldrich, I0634-1MG). After treatment, time-lapse imaging was continued for 10 min and images were acquired every 2 s.

For analysis of calcium-induced mitochondrial fragmentation, iPSC-derived neurons were stained with 100 nM MitoTracker Green FM at day 20 of differentiation. After 1 min of baseline imaging, cells were treated with 5 μM ionomycin and imaging was continued for 20 min with a 1 min interval.

For all live-cell imaging experiments, differentiated neurons were maintained under incubation conditions (37°C, 5% CO_2_ and 80% humidity) during image acquisition. Image analysis was performed with MATLAB 2018b (MATLAB 2018b, The MathWorks, Inc., Natick, MA, USA) or Fiji (68).

### Microscopy and image analysis of the ATP5C1-Rosella reporter (Mitorosella)

The generation, purification and expansion of the smNPC lines carrying the Mitorosella reporter was achieved as previously described (50). Gene-edited smNPCs were further differentiated into neurons following the protocol previously indicated (66).

For the analysis of mitophagy with the Mitorosella reporter, iPSC-derived neurons were seeded on CellCarrier™-96 well microplates (Perkin Elmer, 6005550), and imaging was performed at day 30 of neuronal differentiation. Mitophagy was induced with 25 μM CCCP (Abcam, ab141229) for 24 h. Images were obtained on an Opera QEHS confocal spinning disk microscope (Perkin Elmer) with a 60× water immersion objective (NA = 1.2). Both fluorescent channels for pHIuorin and DsRed were acquired simultaneously.

The automated image analysis was performed as previously described (50).

### Immunocytochemistry

For co-localization analysis of mitochondria and ER, iPSC-derived neurons were seeded on glass coverslips and fixed at day 30 of neuronal differentiation with 4% paraformaldehyde (PFA) for 15 min. Organelles of interest were labeled with antibodies against Tom20 (Santa Cruz Biotechnologies, sc-17 764, RRID:AB_628381, dilution 1:500; secondary antibody: goat anti-mouse IgG [H + L] Alexa Fluor 647, Life Technologies, A-21235, RRID:AB_2535804, dilution 1:1000) and Calnexin (Cell Signaling, 2679, RRID:AB_2228381, dilution 1:500; secondary antibody: goat anti-rabbit IgG [H + L] Alexa Fluor 488, Life Technologies, A-11008, RRID:AB_143165, dilution 1:1000).

For the co-localization analysis of mitochondria, ER and Miro1, iPSC-derived neurons were seeded on glass coverslips and fixed at day 30 of neuronal differentiation with 4% PFA for 15 min. Organelles of interest were labeled with antibodies against Tom20 (Santa Cruz Biotechnologies, sc-17 764, RRID:AB_628381, dilution 1:500; secondary antibody: goat anti-mouse IgG2a Alexa Fluor 546, Life Technologies, A-21133, RRID:AB_2535772, dilution 1:1000), Calnexin (Cell Signaling, 2679, RRID:AB_2228381, dilution 1:500; secondary antibody: goat anti-rabbit IgG [H + L] Alexa Fluor 488, Life Technologies, A-11008, RRID:AB_143165, dilution 1:1000) and Miro1 (Sigma Aldrich, WH0055288M1, RRID:AB_1843347, 1:1000; secondary antibody: goat anti-mouse IgG1 Alexa Fluor 647, Life Technologies, A-21240, RRID:AB_141658, 1:1000).

Image analysis was performed with MATLAB 2018b (MATLAB 2018b, The MathWorks, Inc., Natick, MA, USA).

### Statistical analysis

Statistical analysis was determined using GraphPad Prism 8.1 software (GraphPad Prism version 8.1.0 for Windows, GraphPad Software, San Diego, CA, USA). Statistical tests and *P*-values are defined in the figure legends. To correct for the small sample size used in this study, we used non-parametric tests throughout. All experiments were independently repeated at least three times.

## Supplementary Material

Suppl_Fig1_iPSCcharacterization_200331_ddaa066

Suppl_Fig2_smNPCandDAneuron_characterization_200331_ddaa066
