# Supplementary figures and images for: Impaired mitochondrial–endoplasmic reticulum interaction and mitophagy in Miro1-mutant neurons in Parkinson’s disease
Source: Hum Mol Genet. 2020 Apr 13;29(8):1353–64. doi: 10.1093/hmg/ddaa066 (PMC7254851; doi:10.1093/hmg/ddaa066)

Suppl.Fig. 1

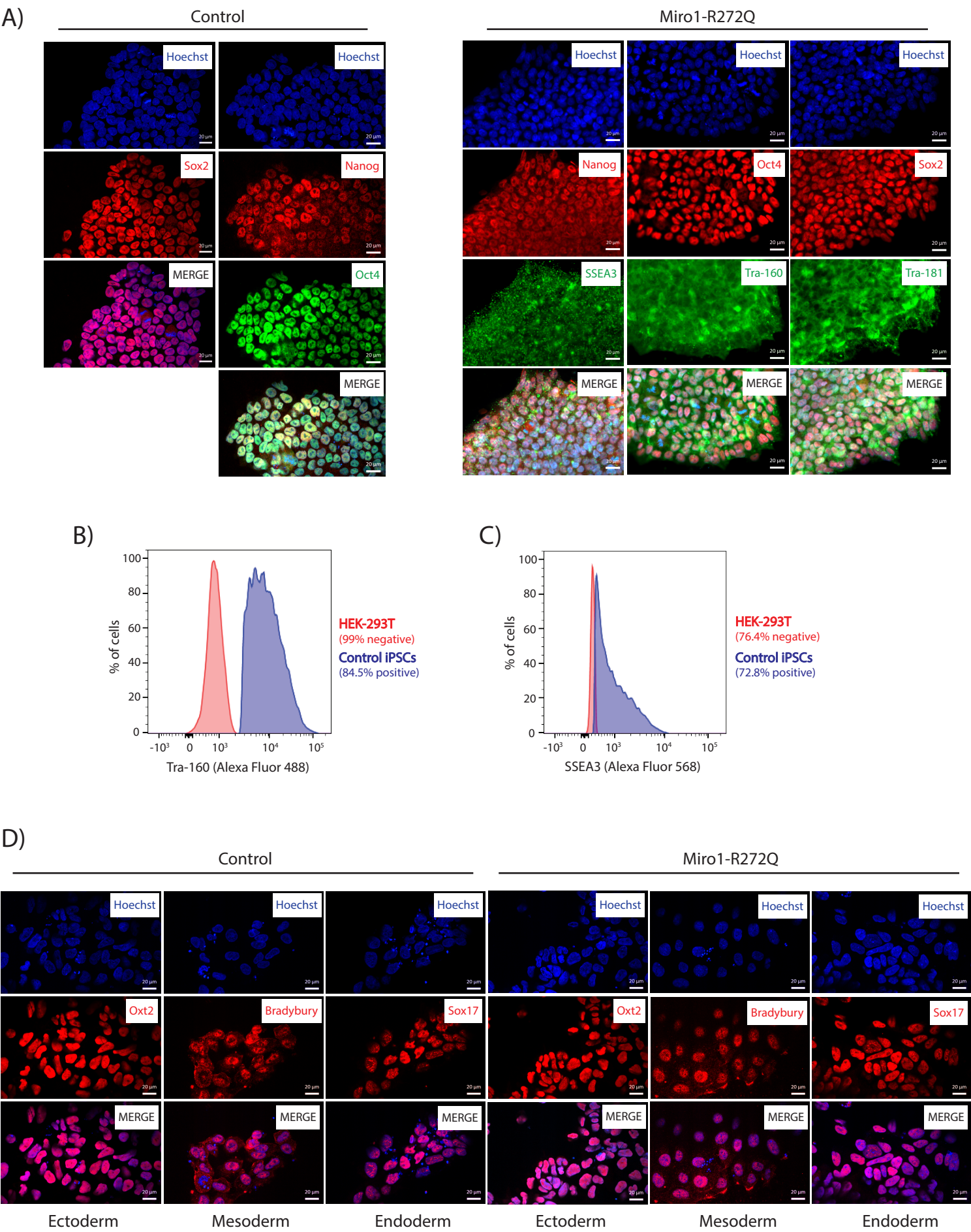

Supplement: Suppl_Fig1_iPSCcharacterization_200331_ddaa066 [file suppl_fig1_ipsccharacterization_200331_ddaa066.pdf]

## Suppl.Fig. 2

A)

Control

Miro1-R272Q

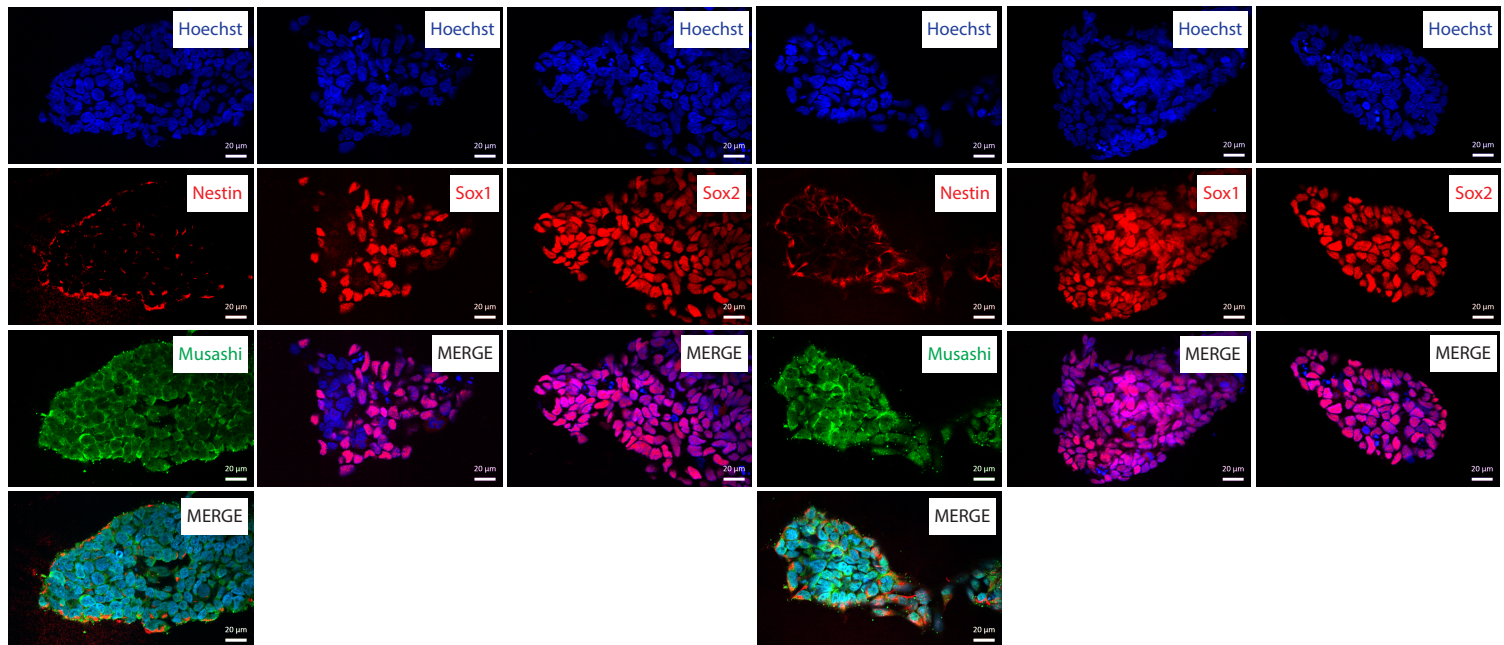

B)

Control

Miro1-R272Q

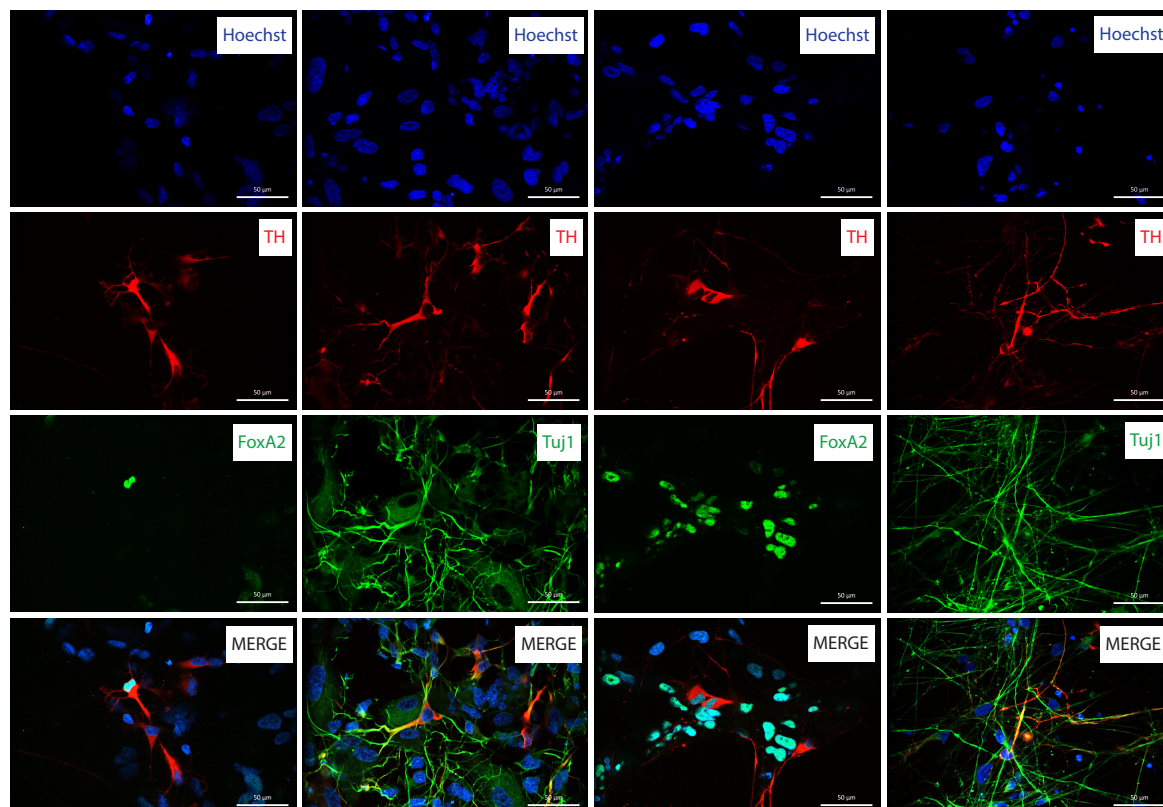

C)

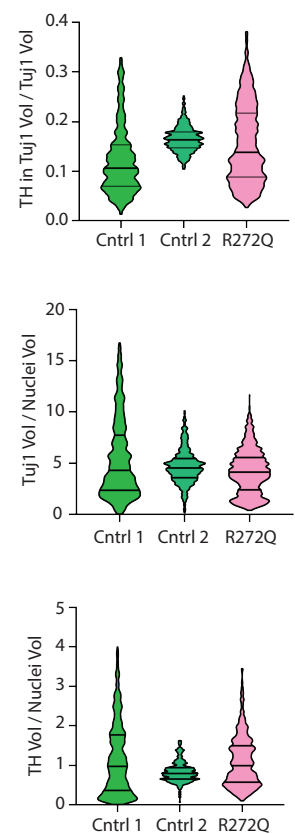

Supplement: Suppl_Fig2_smNPCandDAneuron_characterization_200331_ddaa066 [file suppl_fig2_smnpcanddaneuron_characterization_200331_ddaa066.pdf]
